# Associations of Treatment Outcome Expectations and Pain Sensitivity after Cervical Spine Manipulation in Patients with Chronic Non-Specific Neck Pain: A Cohort Study

**DOI:** 10.3390/healthcare12171702

**Published:** 2024-08-26

**Authors:** Danai Paleta, Stefanos Karanasios, Nikolaos Diamantopoulos, Nektarios Martzoukos, Nikolaos Zampetakis, Maria Moutzouri, George Gioftsos

**Affiliations:** 1 Physiotherapy Department, University of West Attica, 12243 Athens, Greece; 2 Physiotherapy Department, Hellenic Orthopedic Musculoskeletal Training (OMT) eDu, 11631 Athens, Greece

**Keywords:** chronic mechanical neck pain, expectations for treatment, pain perception, pressure pain threshold, manual therapy, manipulations

## Abstract

(1) Background: This cohort study aimed to evaluate the effect of patients’ treatment expectations on pain perception changes following manual therapy cervical manipulations in individuals with chronic mechanical neck pain. (2) Methods: Demographic data were collected by 56 subjects who were asked to fill out the Neck Disability Index (NDI) and the Expectations for Treatment Scale (ETS). All patients received one single cervical manipulation, and pressure pain thresholds (PPTs) were measured before and immediately after the manipulation with a digital algometer. (3) Results: A total of 56 patients participated. Most subjects (62.5%) had high treatment expectations according to the ETS scale. Statistically significant increases in PPTs were noted both locally and in remote areas (*p* < 0.05), with 37.5–48.2% of participants showing clinically significant changes in pain perception. However, no statistically significant correlation was found between high treatment expectations and increased PPTs (*p* > 0.05). (4) Conclusions: Although a significant reduction in pain perception was observed, it did not correlate with patients’ treatment expectations. Future research for further investigation of this hypothesis by comparing real versus sham treatment and exploring additional mechanisms affecting changes in PPTs after cervical manipulations in this population will contribute to a better understanding of the research question.

## 1. Introduction

Neck pain is a common musculoskeletal condition in clinical practice, with one-year prevalence estimates of up to 50% [1,2]. Despite the optimal management of this condition, in a significant proportion of patients, it becomes chronic (duration of symptoms > 12 weeks), accounting for 1.7–11% of the general population [3,4]. Neck pain etiology is usually multifactorial, and a direct pathoanatomic cause is usually non-definable; therefore, this condition is classified as mechanical or non-specific [5,6]. Chronic non-specific neck pain (NSNP) appears to cause significant disability and a decline in quality of life, associated with an increased healthcare burden [7,8,9]. The most effective therapeutic approaches for chronic NSNP may include therapeutic exercises, manual therapy and acupuncture/dry needling (or a combination) that were found to reduce pain and disability at a short- and long-term follow-up [10,11,12,13].

Manual therapy includes thrust joint manipulation and non-thrust mobilization techniques to the joints and/or the related soft tissues [14,15]. Manipulation refers to techniques involving high-velocity, low-amplitude (HVLA) thrusts, while mobilization involves lower-velocity movements [14]. Evidence suggests that cervical spine manipulation can be an effective approach to increase the range of motion, decrease pain and improve functionality in patients with chronic NSNP [16,17,18]. The effectiveness of cervical spine manipulation has been attributed to the interaction of multiple complementary mechanisms [19]. Based on the available research data, spinal manipulation stimulates paraspinal muscle reflexes and motor neuron excitability changes [20,21]. Significant effects have also been recorded in reflex neural outputs to both muscle and visceral organs. Most importantly, spinal manipulations have shown changes in central sensory processing by removing subthreshold mechanical or chemical stimuli from paraspinal tissues [20,22]. Hence, according to different reports, cervical spine manipulation results in significant changes in pain perception in patients with NSNP and healthy individuals immediately after the intervention [14,23,24,25].

Among the specific and non-specific effects of spinal manipulation, the placebo effect is considered to be a significant factor of the treatment outcome of spinal manipulation [26,27]. The placebo effect is generated from previous experiences, beliefs and the clinical context (i.e., the information provided, clinical setting and interaction with the healthcare provider) [26,28]. Treatment expectation is recognized as a fundamental mechanism behind the placebo effect [29]. Notably, patients with NSNP and positive treatment expectations show a more successful outcome than individuals with lower expectations from spinal manipulation [30]. Also, positive treatment expectations were found to produce significant increases in pressure pain thresholds (PPTs) after spinal manipulation in the lower back in healthy individuals [22]. To the best of our knowledge, similar studies investigating the correlation between the treatment outcome expectations and the changes in pain sensitivity in patients with neck pain are lacking.

Therefore, the aim of our study was to investigate the association between treatment outcome expectations and changes in PPTs in patients with chronic NSNP immediately after a cervical spine manipulation and our hypothesis that higher treatment expectations would be positively associated with PPT increases.

## 2. Materials and Methods

### 2.1. Study Design

We conducted a prospective cohort study design adhering to the Strengthening the Reporting of Observational Studies in Epidemiology (STROBE) recommendations [31]. This study was performed in a private physiotherapy clinic in Athens, Greece and took place between April 2023 and September 2023 after approval by the Ethics Committee of the University of West Attica (ID: 40758/25 April 2023). Participants were recruited via social media and verbal communication in the University of West Attica and through referrals in physiotherapy clinics located in the Attica region. Before participation, all participants signed an informed consent form in accordance with the World Medical Association Declaration of Helsinki (2013). All patients were treated by the same physical therapist (ND), who had a 2-year postgraduate training in orthopedic manipulative therapy and 22 years of clinical experience.

### 2.2. Participants

Patients were eligible to participate in this study if they were between 18 and 60 years of age and presented symptoms of NSNP with or without unilateral upper-extremity symptoms lasting more than 12 weeks [32]. Patients were excluded if they had any of the following criteria: resting blood pressure greater than 140/90 mmHg, prolonged history of steroid use, cervical vertebrae fracture or dislocation, whiplash injury, tumor/malignancy, Down’s syndrome, bilateral upper-extremity symptoms, osteoporosis, surgery on the cervical spine, cervical artery dysfunction or known neurologic or rheumatic conditions [30,33].

### 2.3. Procedures

At the initial eligibility assessment, potential participants were examined by a musculoskeletal physiotherapist (SK) with 17 years of clinical experience. After their consent was obtained, the examiner (SK) recorded demographic characteristics, such as sex, age, height, weight, duration of symptoms and previous experience of manual therapy interventions. Also, the patients’ pain intensity during neck movements was recorded using a numeric pain-rating scale from 0 (no pain) to 10 (worst pain). Subsequently, the patients filled the Expectations for Treatment Scale (ETS) questionnaire and the Neck Disability Index (NDI) questionnaire.

The treating physical therapist carried out a history and physical examination procedure, including palpation, special tests and active/passive movements of the cervical spine. The examination procedure adhered to the recommendations of the international IFOMPT cervical framework potential of vascular pathologies of the neck prior to musculoskeletal interventions [33]. Then, the patients were familiarized with the procedures to be used for testing PPTs at local and remote sites. The measurements of PPTs were taken three times by an independent assessor (DP) for each point, and the average value was recorded.

### 2.4. Interventions

**Figure 1:**
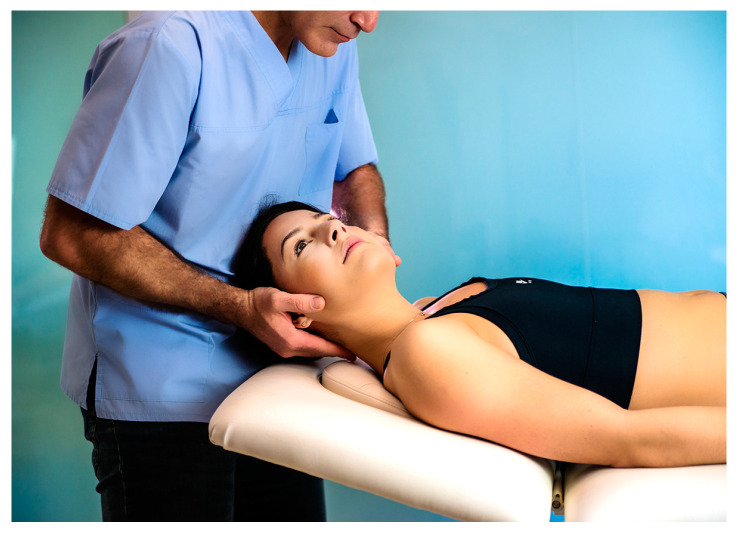
Cervical spine (C4–C5) high-velocity, low-amplitude (HVLA) rotational thrust manipulation for a restricted left-side flexion.

During the intervention, the patients were lying down in a relaxed supine position, and the treating physical therapist applied a high-velocity, low-amplitude (HVLA) thrust at the C4–C5 spinal level towards the most painful side (Figure 1). An attempt was considered to be successful when an ‘audible popping’ followed the technique, and a maximum of two HVLA thrusts were attempted for each participant, if needed. Immediately after completing the intervention, the PPTs were measured 5 min after the intervention by the same assessor (DP).

### 2.5. Outcomes

Patients’ outcome expectation for the spinal manipulation treatment effectiveness was assessed using the ETS. The ETS consists of 5 items that aim to capture patients’ expectations regarding a positive or negative effect of a treatment on their health status [34]. Each item is scored on a 4-point scale ranging from 1 (partially disagree) to 4 (definitely agree) [34,35]. The total score of the scale can range between 5 and 20 points, and higher values indicate higher treatment expectations. The Greek version of the ETS has presented a high level of content validity, an acceptable internal consistency (Cronbach’s alpha: 0.84) and an excellent test–retest reliability (intraclass correlation coefficient: 0.96) about manual therapy in patients with musculoskeletal conditions [36].

The NDI is a widely used and validated outcome measure that aims to determine the self-reported disability in patients with NSNP [37]. Patients are instructed to choose answers that better describe their condition in 10 items of various activities and pain. The score of each item can range from 0 (no pain and no functional limitation) to 5 (worst pain and maximal limitation), resulting in a total NDI score between 0 (no disability) and 50 (totally disabled) [37]. The Greek version of the NDI has shown an excellent internal consistency (Cronbach’s alpha: 0.85) and test–retest reliability (intraclass correlation coefficient: 0.93) [38].

For the measurements of PPTs, a 1 cm diameter hand-held digital algometer (Baoshishan ZP-1000 N 20/22806, Baoshishan, Shenzhen, China) was used following a documented process with excellent intra-rater reliability [39]. The patient was seated with his/her arms resting on the thighs and the hips flexed at 90°. All measurements were conducted starting from the dominant to the non-dominant side in the following order: at the transverse process of the C4–C5 vertebrae, the midpoint of the anterior surface of the biceps brachii muscle and the anterior tibialis muscle (5 cm from the tibial tubercle). The same sequence was followed for the post-intervention process. All measurements were taken by the same blinded assessor (DP) using a metronome pacing the rate of pressure application. When the pressure stimulation became painful during the administration, the patients were instructed to respond with a verbal notification (“now”). The mean value of three measurements was recorded in kg/cm^2^ at each site. The patients rested for 20–30 s between administrations. Pressure algometry has shown excellent reliability (intraclass correlation coefficient: 0.91), with a minimally detectable change (MDC) for the PPT over transverse process of the C4–C5 vertebrae and tibialis anterior muscle of 0.48 and 0.99 kg/cm^2^, respectively [40,41].

### 2.6. Blinding

To limit the influence of bias in the present study, the outcome assessor who assessed the PPTs as well as the treating physical therapist were blinded to the participants’ ETS scores; they did not receive information about the patients’ expectations for the treatment, and they were in different rooms throughout the study. The therapists adhered strictly to this protocol, avoiding any discussion of treatment expectations with the patients. This approach might underlie some ethical concerns, as the therapeutic relationship between therapist and patients, which is crucial for appropriate patient care, was not facilitated. However, the current process ensured the integrity of the results and the scientific rigor of this study.

### 2.7. Sample Size

Based on previous studies that used cervical spine manipulation in patients with NSNP [25,42], a sample size calculation (power 0.80, significance level 0.05) was conducted by using changes in PPTs as a primary outcome measure with the StatCalc application of Epi Info™ (Version 7.2.5.0). A sample size of 37 was estimated to be sufficient to detect an effect size of 1 on PPTs of the cervical spine, according to prior similar studies.

### 2.8. Statistical Analysis

A normality test of the data was performed using the Shapiro–Wilks test. The participants’ characteristics, such as sex ratio, age, BMI, questionnaire scores, duration of symptoms and previous experience in manual therapy application, were presented with descriptive statistics. Normally distributed data were presented with means and standard deviations (SD) and were analyzed with parametric tests, while data without normal distribution were presented with median values and interquartile ranges (IQR) and were analyzed with non-parametric tests.

The changes in PPTs at each anatomical region between pre- and post-intervention were analyzed using the Wilcoxon signed rank test for paired observations. The associations between changes in PPTs with sex and previous experience in manual therapy interventions were calculated with Pearson chi-square tests. Spearman’s Rho correlation coefficients were used to investigate the association between changes in PPTs and ETS, pain intensity, NDI, age, BMI or symptom duration. Spearman’s correlation coefficient was classified as weak (≤0.39), moderate (0.4 to 0.69) or strong (0.7 to 1.0) correlation [43]. Also, changes in PPTs between patients with high (ETS score > 12) and low (ETS score ≤ 12) treatment outcome expectations were analyzed using the Mann–Whitney U test. All data were analyzed with IBM SPSS (Version 25), and the level of significance was defined at 0.05.

## 3. Results

Of the sixty-three patients with NSNP that were assessed for eligibility, seven patients were excluded due to osteoporosis (5), rheumatoid arthritis (1) and a history of neck fracture (1). Hence, 56 patients (35 women and 21 men) with a median age of 31.5 (25–75% IQR: 24, 44.75) years were included in this study. The median duration of symptoms was 24 (25–75% IQR: 6, 96) months. Among the total sample, thirteen patients (23.2%) with NSNP had a previous experience in manual therapy cervical manipulation, while the remaining 43 (76.8%) received the current intervention for the first time. The patients’ demographic characteristics are shown in Table 1.

The eligible participants presented a median pain intensity score of 2.5 (25–75% IQR: 1.5, 4.95) and a median NDI score of 10.5 (25–75% IQR: 7, 14). The median ETS score was 13.5 (25–75% IQR: 11.25,15), with the majority of the eligible patients (62.5%) demonstrating high expectations (>12.5 ETS score) for the cervical manipulation.

There was a statistically significant increase in PPTs between pre- and post-intervention at both sides of the C4–C5 transverse process (z_right_ = −5.46, *p* < 0.001; z_left_ = −5.3, *p* < 0.001), biceps brachii (z_right_ = −4.26, *p* < 0.001; z_left_ = −4.32, *p* < 0.001) and tibialis anterior (z_right_ = −6.01, *p* < 0.001; z_left_ = −5.58, *p* < 0.001). The median values and the ranges of the PPTs before and after the cervical manipulation at each body site are shown in Table 2.

There was no statistically significant correlation between the ETS scores and the changes in PPTs at C4–C5, bicep brachii and the right side of the tibialis anterior. A statistically significant ‘weak’ correlation was found between the ETS scores and the changes in PPTs in only the left tibialis anterior (r = 0.29, *p* = 0.02). A statistically significant ‘weak’ correlation was also found between the patients’ symptom duration and the changes in PPTs in the right side of the C4–C5 vertebra (r = −0.26, *p* = 0.45), the bicep brachii (r = −0.30, *p*_right_ = 0.65; r = −0.37, *p*_left_ = 0.94) and the right side of the tibialis anterior (r = 0.29, *p* = 0.02). Similarly, a weak correlation was found between the pain intensity (0–10) at baseline and changes in PPTs in the right side of the C4–C5 vertebra (r: −0.28, *p* = 0.033). No significant correlations were found between the changes in PPTs and the participants’ age, BMI or NDI scores at all measurement sites. The results of Spearman’s correlations Rho are shown in detail in Table 3.

The changes in PPTs between patients with high treatment expectations (ETS score > 12.5) and those with low treatment expectations (ETS score < 12.5) showed no statistically significant changes both at local and distal body sites of measurements. Table 4 includes the median values (25–75% IQR) of the change in PPTs in each group at all sites of measurements. Our findings showed that women patients with NSNP presented a statistically significant correlation with high treatment expectations (x^2^ = 8.53, *p* = 0.005), while there was no statistically significant correlation between previous experience in MT treatment and high or low treatment outcome expectations (x^2^ = 1.55, *p* = 0.33).

## 4. Discussion

The main findings of our study indicate that an HVLA thrust on the cervical spine produces an immediate hypoalgesic effect both at local and distal sites in patients with chronic NSNP. Most importantly and opposed to our hypothesis, the significant increases in PPTs were not correlated with patients’ high treatment outcome expectations as measured with the ETS questionnaire.

The majority of patients with NSNP included in our study presented high treatment expectations (62.5%). Comparatively, a similar proportion of patients (60–75%) with neck and low back pain have been reported to present specific expectations of benefit from spinal manipulation [30,44,45]. As patients’ expectations are considered to be a fundamental mechanism behind the placebo effect, patients’ beliefs seem to shape treatment outcomes [46]. Several studies indicate that patients with higher expectations of benefit experience greater symptom relief from spinal manipulation [47,48]. However, the variability in study methodologies, outcomes and the way placebo effects are measured may lead to inconsistent conclusions about the relationship between the efficacy of spinal manipulation and the placebo effect [49]. According to earlier research, there are contradictory results for the predictive value of positive expectations for spinal manipulation in patients with NSNP [45,50]. Two reports identified a predictive value of patient expectancies on recovery [30,51]. Similar to our findings, other studies failed to establish an association between treatment expectancies and outcomes [44,50]. This controversy can be potentially attributed to the variable measurement tools to capture treatment outcome expectations among the studies, including a seven-point Likert scale (1 = complete relief from symptoms such as pain, stiffness, swelling, numbness, weakness, instability and 7 = to prevent future disability) or a numerical rating scale from 0 (‘Not at all likely that I will be completely recovered’) to 10 (‘Very likely that I will be completely recovered’). Treatment expectations are considered to take different forms, such as the predicted expectations (what a patient believes will occur), ideal expectations (what a patient wants to occur), normative expectations (what a patient believes should occur) and unformed expectations (the lack of bias for the result). Hence, the interpretation of each study’s findings regarding the predictive value of patient expectancies on recovery should be interpreted with caution, considering the lack of validity of the instruments used to measure these expectations. Based on our knowledge, this is the first study using a validated questionnaire (ETS) to measure patients’ expectations for manipulative therapy [36].

The significant decrease in mechanical pain sensitivity found following the cervical spine manipulation was in line with previous studies evaluating patients with neck pain and healthy individuals [14,23,52,53]. Notably, the observed changes in PPTs at C4–C5, biceps brachii and tibialis anterior exceeded the expected MDCs, suggesting that the current intervention can potentially provide meaningful changes in patients with NSNP [23,40,41]. However, the statistically significant differences in pressure algometry should be interpreted with caution since they do not necessarily represent clinically important differences in outcomes within time. Some authors proposed a change between 0.5 and 3.4 kg/cm^2^ (or 20–50% change) to be a clinically important difference for PPTs [41,54,55]; nevertheless, these values require further validation in chronic patients with NSNP by comparing the changes with clinically relevant outcome measures.

Several neurophysiological mechanisms have been proposed to explain the improvements in pain perception after cervical manipulations [56,57,58]. Most of them have been attributed to neuroplastic changes that may result in an inhibition within the dorsal horn that can potentially decrease sensitization [19,57,58,59]. Also, an activation of both segmental and central descending inhibitory pain pathways can explain the local and widespread improvements in PPTs and pain perception, respectively [59,60,61,62]. In addition to these mechanisms, Bialosky et al. (2009) emphasized the critical role for non-specific effects, such as the treatment outcome expectation associated with spinal manipulation [22]. Contrary to the previous suggestion, our results indicated that the changes in mechanical pain sensitivity after the cervical manipulation were not related to the patients’ treatment outcome expectation. Possibly, this discrepancy between the study findings can be explained due to the sample characteristics (chronic patients with NSNP versus healthy individuals) and other methodological variations, such as the expectation intervention, the type of spinal manipulation or the type of pain algometry.

Different confounding factors have been reported to influence the role of expectations in recovery for patients with neck pain, such as previous experiences, previous episodes of pain, disability level, duration of symptoms, pain intensity and depression [44,45,63,64]. Notably, previous experience in treatment with manual therapy was not associated with high or low treatment outcome expectations. Also, despite that our findings indicated significant correlations between changes in pain sensitivity after the intervention and symptom duration or pain intensity, these associations were weak (r < 0.39) and should be interpreted with caution. It should be noted that an individual’s psychological state is considered a critical associated factor influencing the role of expectancies on recovery of patients with neck pain. Psychological conditions, such as depression, anxiety and stress, may affect pain perception, treatment outcomes and success in most chronic musculoskeletal pain conditions [65]. Nevertheless, the role of psychological state was not evaluated in the present study, and further research is required to examine this possible confounding factor.

### Limitations and Future Research

Our study should be interpreted in the light of some limitations. First, we decided to include a single experimental session rather than a series of sessions, and the changes in PPTs may differ over a course of sessions. Second, the clinical interpretation of our findings requires a careful consideration of the participants’ characteristics, such as the mild pain intensity and mild to moderate disability. Also, our sample consisted mostly of women with a median age of 31.4 years, which is not representative of the general population suffering from chronic NSNP. Third, although we used a cut-off ETS score of 12.5 to distinguish between high and low treatment expectations, further investigation is required to confirm this hypothesis. Last, the success of blinding the assessors and the therapist was not assessed in our study.

We examined the correlations between treatment expectations and changes in pain sensitivity after cervical manipulation and also investigated the impact of several confounding factors, including age, duration of symptoms, disability, previous experience and pain intensity. However, the observational nature of our study limits the opportunity to determine cause-and-effect relationships because of the absence of a control group or a sham-treatment group. Therefore, further research using an experimental design such as randomized controlled trials comparing real versus sham treatments would be valuable to investigate the effect of expectation on pain sensitivity.

## 5. Conclusions

Our study revealed that, although an HVLA thrust on the cervical spine induced an immediate hypoalgesic effect in patients with chronic NSNP, these changes did not significantly correlate with the patients’ treatment expectations. The majority of patients in our study demonstrated high treatment expectations; however, the association between these expectations and the actual treatment outcomes remains complex and inconclusive. The observed changes in pain sensitivity after the intervention exceeded the expected MDCs both at local and distal sites; however, further research using an experimental design is required to evaluate the clinical importance of these changes.

## Figures and Tables

**Table 1:** Demographic and clinical characteristics of patients with non-specific neck pain (N = 56).

Characteristic	Median or No (Percentage)	Range	25–75% IQR
Sex			
Women	35 (62.5%)		
Men	21 (37.5%)		
Previous experience in cervical manipulation			
Yes	13		
No	43 (76.7%)		
Age (years)	31.5	19–55	24–44.75
BMI (kg/m^2^)	23.9	16–34.8	21.4–27.5
Pain intensity (0–10)	2.5	1–7.5	1.5–4.9
NDI	10.5	2–28	7–14
ETS	13.5	7–20	11.25–15
High treatment expectations	35 (62.5%)		
Low treatment expectations	21 (37.5%)		

Abbreviations: N, sample; kg/m^2^, kilograms. Meters^2^; NDI, Neck Disability Index; ETS, Expectation Treatment Scale; IQR, interquartile range.

**Table 2:** Pressure pain thresholds (kg/cm^2^) at baseline and after the HVLA thrust at local and distal sites of the body.

	Pre-Intervention PPT ^a^	Post-Intervention PPT ^a^	*p*-Value ^b^
C4–C5 (right)	3.33 (2.35–4.73)	3.96 (2.85–5.64)	<0.001
Range	1.30–7.43	1.53–9.76	
C4–C5 (left)	3.25 (2.33–4.88)	3.65 (2.74–5.75)	<0.001
Range	1.30–7.86	1.56–9.36	
Biceps brachii (right)	4.10 (2.68–5.65)	4.75 (3.30–6.22)	<0.001
Range	1.46–10.60	1.60–12.40	
Biceps brachii (left)	4.03 (2.80–5.76)	4.65 (3.11–6.55)	<0.001
Range	1.26–11.23	1.40–12.56	
Tibialis anterior (right) Range	9.01 (5.66–14.43) 3.76–21.86	11.73 (7.00–16.99) 4.13–22.46	<0.001
Tibialis anterior (left) Range	8.96 (5.44–14.06) 3.43–24.46	11.31 (6.75–17.03) 3.83–25.26	<0.001

^a^ Values are expressed as median (25–75% IQR). ^b^ Wilcoxon signed rank test for paired observations, pre-intervention versus post-intervention measurements. Abbreviations: kg/cm^2^, kilograms. centimeters^2^; IQR, interquartile range; HVLA, high-velocity low-amplitude thrust.

**Table 3:** The association between changes in PPTs (kg/cm^2^) at each site and the Expectations Treatment Score, Neck Disability Index, symptoms duration, pain intensity, age and body mass index as examined with Spearman’s correlation.

	ETS	NDI	Age	Symptom Duration	Pain Intensity	BMI
C4–C5 (right)	r = 0.94, *p* = 0.49	r = −0.13, *p* = 0.31	r = 0.24, *p* = 0.07	r = 0.01, *p* = 0.09	r = −0.28, *p* = 0.03	r = −0.03, *p* = 0.31
C4–C5 (left)	r = −0.10, *p* = 0.94	r = −0.22, *p* = 0.09	r = 0.62, *p* = 0.65	r = 0.26, *p* = 0.04	r = −0.24, *p* = 0.07	r = −0.32, *p* = 0.16
Biceps brachii (right)	r = 0.06, *p* = 0.65	r = −0.002, *p* = 0.98	r = 90, *p* = 0.98	r = 0.30, *p* = 0.02	r = −0.11, *p* = 0.39	r = 0.01, *p* = 0.46
Biceps brachii (left)	r = −0.09, *p* = 0.94	r = −0.09, *p* = 0.49	r = −0.13, *p* = 0.34	r = 0.37, *p* = 0.004	r = −0.17, *p* = 0.19	r = 0.01, *p* = 0.98
Tibialis anterior (right)	r = 0.10, *p* = 0.43	r = −0.16, *p* = 0.22	r = −0.11, *p* = 0.42	r = 0.28, *p* = 0.03	r = −0.15, *p* = 0.24	r = −0.07, *p* = 0.57
Tibialis anterior (left)	r = 0.29, *p* = 0.02	r = −0.06, *p* = 0.61	r = −0.16, *p* = 0.22	r = 0.24, *p* = 0.06	r = −0.15, *p* = 0.26	r = −0.01, *p* = 0.98

Abbreviations: r, correlation coefficient; *p*, *p*-value; kg/cm^2^, kilograms. centimeters^2^; ETS, Expectations Treatment Score; NDI, Neck Disability Index; BMI, body mass index.

**Table 4:** Pressure pain thresholds (kg/cm^2^) between pre- and post-HVLA thrust at local and distal sites of the body in patients with high and low treatment expectations.

	Patients with High Treatment Expectations	Patients with Low Treatment Expectations	Mann–Whitney U ^b^	*p*-Value ^b^
	PPT Pre–Post ^a^	PPT Pre–Post ^a^		
C4–C5 (right)	0.43 (0.26, 1.03)	0.60 (0.08, 1.26)	359.500	0.89
C4–C5 (left)	0.53 (1.33, 0.90)	0.70 (0.36, 1.85)	277.000	0.12
Biceps brachii (right)	0.43 (0.00, 1.23)	0.90 (−0.10, 1.46)	317.000	0.39
Biceps brachii (left)	0.53 (−0.13, 0.70)	0.83 (−0.03, 1.35)	297.500	0.23
Tibialis anterior (right)	1.30 (0.80, 2.90)	1.56 (0.63, 3.51)	349.500	0.76
Tibialis anterior (left)	1.70 (0.80, 2.46)	0.70 (−0.08, 2.95)	300.000	0.25

^a^ Values are expressed as median (25–75% IQR). ^b^ Mann–Whitney U test, between group comparison of the change in PPTs at each site of measurement. Abbreviations: kg/cm^2^, kilograms. centimeters^2^.

## Data Availability

The raw data supporting the conclusions of this article will be made available by the authors on request.
